# Monthly Summary

**Published:** 1867-11

**Authors:** 


### MONTHLY SUMMARY.

*Animal Grafts.*—A French naturalist, M. Vulpian, cut off the tails of tadpoles, and saw them not only live but *grow* for ten days, indifferent to all theories of nervous centres, digestive apparatus, or circulatory systems. But the member that seems to have the strongest dose of the "vital principle," is the tail of a rat. The following experiment was made by Mr. Bert. He dried a rat's tail under the bell of an air pump, and in immediate proximity to concentrated sulphuric acid, so as gradually to deprive it of all moisture. Then he placed it in a hermetically sealed glass tube for five days. At the end of this time he subjected it for a number of hours to a temperature of 98 degrees centigrade in a stove, and subsequently sealed it a second time in his tube. Four days more having elapsed, he united this tail by its cut extremity, to the freshly cut stump of a living healthy rat, and quietly awaited the result. His success was as complete as it was marvellous. It commenced to expand and perform the natural duties of a tail, and three months afterwards, he demonstrated by a second amputation, and a careful injection, that it was furnished with proper vessels and was a living part of the second rat!

What rich lessons practical surgery may learn from such experiments, can be imagined. A careful anatomist has transplanted a fragment of bone from the skull of one rabbit to the skull of another, and found it form adhesions and replace the lost portion perfectly. A piece of periosteum taken from a rabbit twenty-four hours after death, grew and produced bone when grafted neatly on a living animal of the same species. Nerves also have been removed from one body to another with success, and some very singular results noticed were a portion of a motor was excised and supplied by a fragment of a sensory filament. The disease to which grafted members are subject, after they have been exposed to certain reagents, are also full of hints for the pathologist and the physician.—*Med. and Surg. Reporter.*

---

*The Prudent Live Longest.*—In a very careful and laborious *Appendix to the Eighteenth Annual Report of the Prudential Assurance Company*, by Henry Harben, Esq., is given the experience of the Company in the industrial branch for the years 1864, 1865, and 1866; and the author ingeniously compares the Company's statistics with those issued by the Registrar General. The experience is this: that among the artisan and small tradesmen class of lives, the numbers exposed to risk were in the proportion of 48.3 male to 51.7 female, in this respect assimilating to the proportions of the general population of England and Wales; that the rate of mortality during these three years was 21.67 per 1000 whereas, in all England and Wales it was 23.63 —the difference in favor of the Prudential Company being 1.96 per 1000. Since it is the most prudent of the working classes who insure their lives, these facts, brought prominently forward by Mr. Harben, tend to verefy the old saw, that "the prudent live longest."—*Brit. Med. Journal.*

---

*New Lingual Muscle.*—Bochdolek, Jr., describes a new small muscle of the tongue, extending longitudinally in the middle line between the two genio-hyo-glossi—*Reinhert und Du Bois Raymond's Archiv*, 1866, from *Journal Anatomy and Physiology*, No. II.

*The Preservation of Sulphate of Iron.*—Signor Pavisi recommends the following method of preserving sulphate of iron from oxidation. Mix four parts of pure crystallized sulphate of iron, and an equal quantity of finely powdered gum arabic, with distilled water, and evaporate the solution in a water bath, at a low heat, till it has a sufficient consistency to be poured out on plates of glass. When it has been poured out in this way, and allowed to dry at a temperature of 38° cent. in the dark, it may be cut up into lozenges, which can be kept for any length of time in a coloured stoppered bottle.—*London Lancet.*

---

*Excretion of Urea.*—The *American Journal of the Medical Seiences* for October, publishes a very clever inaugural thesis by Dr. T. R. Noyes, containing a record of experiments on four persons, to determine the effect of food, sleep and exercise in the excretion of urea. The first week the parties experimented on used a mixed diet; the second week they lived exclusively, on animal food, the third on purely vegetable food with the exception of a little milk in their bread, their tea and their coffee, while during the fourth week, the diet was the same as the third, but the subjects of the experiment took an unusual amount of exercise.

The first point noticed is that there is no immediate change in the excretion of urea after an alteration of diet, but that it requires three days to exhibit the full effect. Another fact made known in these experiments is that the old rule for estimating the proportional quantity of urea excreted, by the specific gravity of the urine, is by no means of universal application. As for diet, it was found that animal food increased the excretion of urea 169 per cent. but diminished the weight of the body. Free *uric acid* was detected, showing that the nitrogenous matter had not been all oxidated to urea. On changing to a vegetable diet the urea was diminished 75 per cent. Exercise slightly increased the quantity of urea, but marked increase was observed only as the result of fatigue.

Coffee increased the urea 14 per cent. To determine the influence of *sleep*, our author lay abed all day for a week, during which time, he found that he eliminated 31 per cent. more urea during the day than at night. The effect of mental occupation is not so distinctly shown, but there appeared to be somewhat less urea excreted during active mental work than when the mind was at rest. The author discharged 2.41 per cent. more urea upon light reading than upon arithmetical calculations.

---

*New Researches on the Cardiac Circulation of Animals.*—Dr. JUDEE has just published a pamphlet on this subject. He shows that in frogs what is by common consent, called first movement, is compounded of the auricular portion and the dilatation of the ventricle, and the dilatration, *per contra* of the two auricles with which the heart of this batrachian is provided. In the second part of the book, relying not only on his own experiments on frogs, but on those made on horses by MM. CHAUVEAU and MAREY, M. JUDEE stated that these physiologists have taken for the commencement of the first movement, or systole, was nothing but the end of the second, or diastole of the heart. In other words, that the systole of the auricle does not form part of the systole of the ventricle, but of its diastole; so that, in fact, in the horse, at least, the cardiac revolution does not commence, as is generally supposed, by the systole of the heart, but by its diastole. When M. JUDEE compares this cardiac revolution to a measurement in three movements, he is led to admit: 1. That the first movement, or great silence, corresponds to the dilatation of the ventricle. 2. That the second and third movements are formed by the sounds of the heart separated one from the other by the short silence, during which the ventricle contracts itself.—*Brit. Med. Journal.*

---

*A New Dentifrice.*—Phenic, or as we more commonly call it, carbolic acid, would come into greater use were it a more manageable drug. A specimen of a phenol soap is offered, and is claimed to be of great use in skin diseases, while a perfumed phenol is presented, said to be a really delightful toilet water and dentifrice. The proportions used are ten grammes of the crystallized acid to a litre of water, with various aromas. When used as a dentifrice, a spoonful of this is added to a quart of water. The phenate of soda can be used with great success as an unguent (one part to ten of simple cerate) in parasitic affections and a comb dipped in a solution of it, and passed through the hair is an efficient remedy in pityriasis, etc. Internally it has been administered by inhalation, by injection, and by the stomach. For the latter purpose a solution of one part in a thousand has been employed.—*Medical and Surgical Reporter.*

---

*A Remarkable Invention.*—Most people have a wish to preserve the bodies of deceased friends or relations from the changes of appearance by decay after death as long as possible. Though the eye has ceased to flash, the lips to move, or the voice to give response, we would fain have the clay tenement preserve its natural appearance.

To effect this object, a *perfectly air-tight* burial case has been invented, which has preserved a corpse at Bellevue Hospital for *three months.*

This burial-case, which certainly must supercede all others, is wonderfully simple in its construction, and is closed by means of a *single screw.*—*Med. and Surg. Reporter.*

---

*Mr. Hoff and the New York Academy of Medicine.*—At the last meeting of the Academy of Medicine, the following resolutions were unanimously adopted;

Whereas, W. L. Hoff, proprietor or agent of the "Hoff Malt Extract," is issuing publications through the secular papers, and by means of pamphlets and circulars professing to quote favourable opinions expressed in a report of a committee of the Academy;

And, Whereas, the said Hoff is widely circulating a letter purporting to have been written by a Fellow of this Academy;

And, Whereas, the publications of said Hoff are so adroitly and designedly worded as to impress the mind of the reader with the belief that the Acadamy has endorsed his nostrum, and has thus apparently compromised its dignity and professional standing—Therefore,

Resolved, That the New York Academy of Medicine does hereby proclaim and declare that it has not expressed any opinion in regard to "Hoff's Malt Extract," and that any and every use of its name in reccommending said Extract is unauthorized by the Academy.

Resolved, That a copy of the above preamble and resolutions be sent to the Medical journals of this city, and that the Medical journals throughout the country be requested to publish the same in justice to the Academy and the profession.—*New York Med. Jour.*

---

*A new Method of Resuscitation from Hyperanæsthesia by Chloroform.*—At a recent meeting of the New York Academy of Medicine, Dr. Worster read a case in which chloroform had been administered to a patient, by a party whom he regarded as competent, as a preparatory step to an operation, by himself, for the relief of hæmorrhoids. Suddenly the patient had stertorous breathing, became pulseless, and exhibited all the symptoms of a speedy dissolution; but by the simple expedient of reversing his position, and inclining his body to an angle of forty-five degrees, he was fully restored.—*N. Y. Med. Record.*

---

